# Fifteen Years of Myotonic Dystrophy Type 1 in Mexico: Clinical, Molecular, and Socioeconomic Insights from a National Reference Cohort

**DOI:** 10.3390/genes16121515

**Published:** 2025-12-17

**Authors:** César M. Cerecedo-Zapata, Araceli Guerra-Grajeda, Luz C. Márquez-Quiróz, Paola Arciga-Portela, Rosa E. Escobar-Cedillo, Guadalupe E. Jiménez-Gutiérrez, Óscar A. Pérez-Méndez, Jorge S. Velasco-Flores, Blanca A. Barredo-Prieto, Norberto Leyva-García, Bulmaro Cisneros, Nadia M. Murillo-Melo, Jonathan J. Magaña

**Affiliations:** 1 Laboratory of Genomic Medicine, Department of Genetics, National Rehabilitation Institute (INR-LGII), Mexico City 14389, Mexicovejor17@gmail.com (J.S.V.-F.);; 2 Hospital Epidemiological Surveillance Unit, National Rehabilitation Institute (INR-LGII), Mexico City 14389, Mexico; 3 Department of Bioengineering, School of Engineering and Sciences, Tecnologico de Monterrey, Campus Ciudad de México, Mexico City 14380, Mexicooscar.perez.m@tec.mx (Ó.A.P.-M.); 4 School of Medicine and Health Sciences, Tecnologico de Monterrey, Monterrey 64700, Mexico; 5 Department of Muscular Dystrophy and Electrophysiology, National Rehabilitation Institute (INR-LGII), Mexico City 14389, Mexico; 6 Department of Genetics and Molecular Biology, Center of Research and Advanced Studies-National Polytechnic Institute (CINVESTAV-IPN), Mexico City 07360, Mexico

**Keywords:** myotonic dystrophy type 1, molecular diagnosis, socio-epidemiologic analysis, CTG repeats, Mexican population

## Abstract

**Background/Objectives**: Myotonic dystrophy type 1 (DM1) is a rare, multisystemic disorder caused by an expanded (CTG)n repeat in the *DMPK* gene. Although DM1 has been studied in several populations, access to molecular diagnosis and comprehensive care remains limited in many low- and middle-income countries. This study provides an updated overview of DM1 in Mexico, from diagnostic implementation to patient management, describing key clinical and genetic findings. **Methods**: We conducted a nationwide, 15-year prospective study at Mexico’s National Reference Center for neuromuscular diseases. A total of 853 individuals at risk were subjected to clinical and molecular evaluation using PCR, TP-PCR, and SP-PCR, encompassing symptomatic, pre-symptomatic, prenatal, and preimplantation genetic diagnosis. Socioeconomic, clinical, and molecular variables were analyzed. **Results**: A total of 488 individuals were confirmed as DM1 carriers, with the most prevalent phenotypes being classic (36.5%) and juvenile (28.5%). Genomic analysis revealed a correlation between CTG tract sizes and phenotypes. Intriguingly, interrupted CTG repeat tracts were identified in 2.8% of DM1 carriers, who exhibited milder clinical phenotypes and a reduced degree of somatic and intergenerational instability. Survival analysis revealed a reduction in symptom-free survival in patients with larger expansions, while interrupted CTG tracts were associated with delayed onset. **Conclusions**: The centralization of diagnostic services in Mexico resulted in regional disparities, impacting early diagnosis and family planning. This study highlights the clinical and molecular diversity of DM1 in a Latin American population and underscores the urgent need for decentralized diagnostic services, integrated care models, and tailored prognostic tools in underserved settings.

## 1. Introduction

The Myotonic dystrophy type 1 (DM1) is a dominantly inherited, multisystemic, rare disorder characterized by a highly variable clinical presentation and progressive disease course [1]. Its global incidence is estimated at approximately 1 per 8000 live births, with a prevalence ranging from 5 to 20 per 100,000 individuals, depending on the population studied [2,3]. However, recent studies indicate that the prevalence of individuals carrying CTG repeat expansions in *DMPK* may be up to five-fold higher than previously estimated [4], suggesting that the disorder remains substantially underdiagnosed across multiple populations. DM1 is caused by an abnormal unstable expansion of a (CTG)n trinucleotide repeat in the 3′untranslated region of the *DMPK* gene, which is located on chromosome 19q13.3 [5]. In unaffected individuals, the number of CTG repeats ranges from 5 to 34, while affected individuals carry expansions ranging from 50 to several thousand repeats [6,7,8,9,10]. A well-established inverse correlation exists between repeat length and age at onset, with longer expansions associated with earlier onset and more severe clinical manifestations [11,12]. Intermediate alleles, representing a pre-mutation state, display a range of CTG repeats between 35 and 49, exhibit no clinical significance, but are genetically unstable.

DM1 is recognized as one of the most variable human genetic diseases in terms of its phenotypic expression [1]. While the presence of skeletal muscle symptoms, including myotonia, weakness, wasting, and atrophy, is characteristic of the condition, the disorder frequently involves multiple organ systems, resulting in complications affecting the cardiac, respiratory, endocrine, gastrointestinal, ophthalmologic, and central nervous systems [13,14]. In addition to its physical manifestations, DM1 frequently results in significant psychosocial and socioeconomic consequences, which often lead to substantial impairment in patients’ quality of life and daily functioning [1]. Conventionally, three primary clinical manifestations of DM1 have been delineated based on age of onset and severity of symptoms: congenital, classic, and mild [15]. However, some authors have proposed further subclassification of the classic form into juvenile and adult-onset (classic) subtypes [15]. The congenital form is the most severe, often manifesting in the neonatal period with hypotonia, respiratory failure, and early mortality [16]. Conversely, individuals afflicted with the mild form of the condition typically exhibit a reduced or absent symptomatology and maintain a near-normal life expectancy. Patients afflicted with juvenile or classic forms of the disease often experience progressive multisystem deterioration and reduced survival, thereby further compounding the personal and societal burden of the disease [17,18].

Despite the importance of early detection and comprehensive care, access to molecular diagnostic tools and specialized follow-up remains limited in many regions, particularly in Latin America, Africa, and parts of Asia [19,20,21,22]. In such contexts, the limited availability of resources and the presence of geographic disparities impede the timely identification of DM1 and the subsequent provision of multidisciplinary management. The implementation of efficient diagnostic pathways, including molecular testing, genetic counseling, family-based risk assessment, and longitudinal clinical monitoring, is fundamental to ensure optimal patient care. Furthermore, the advancement of knowledge concerning the clinical and molecular features of DM1 in various ethnic and geographic populations is of paramount importance for the development of precision interventions and the direction of future therapeutic research.

The aim of the present study provides a comprehensive characterization of DM1 in a Latin American population by examining clinical, genetic, demographic, and epidemiological variables in a large Mexican cohort. This was achieved through the implementation of diagnostic procedures and multidisciplinary management at a national reference Center in Mexico, providing an updated overview of the disease and longitudinal follow up over a fifteen-year period. As the sole publicly accessible diagnostic center for DM1 in Mexico, our institution offers a unique opportunity to evaluate a representative national sample and identify trends in disease burden, diagnostic access, and phenotypic diversity. Thus, this work describes the natural history and prognosis of DM1 in this underrepresented population. Moreover, the research is also to highlight key insights that may inform the design of long-term clinical trials and contribute to improving care delivery in similarly underserved regions worldwide.

## 2. Materials and Methods

### 2.1. Subjects of Study

The study cohort comprised 853 individuals, including 488 subjects with a confirmed diagnosis of DM1 and their healthy at-risk relatives. All subjects were screened for the DM1 mutation through molecular testing performed at the National Rehabilitation Institute–Luis Guillermo Ibarra Ibarra (INR-LGII) in Mexico City. Index cases underwent comprehensive clinical evaluation to identify symptoms related to DM1, complemented by electrophysiological studies conducted using the Nicolet Biomedical Viking IV system. (Nicolet Biomedical Inc., Wimberley, TX, USA) Prior to enrollment, written informed consent was obtained from all participants. The study protocol received approval from the Institutional Research and Ethics Committee of the INR-LGII (INR-28/09) and was conducted in full accordance with the Declaration of Helsinki and relevant ethical guidelines. In addition, a standardized questionnaire was administered to collect sociodemographic data, including education level, occupation, household income, and disease-related expenditures. These data were then compared with official statistics from the National Institute of Statistics and Geography of Mexico (INEGI) to contextualize the socioeconomic impact of DM1 on the affected population.

### 2.2. DM1 Testing Programs

The implementation of an initial symptomatic diagnosis protocol was undertaken to facilitate specialized, multidisciplinary medical care with the aim of reducing the psychosocial burden associated with DM1. A wide array of clinical and molecular data was collected from various regions across Mexico. The multidisciplinary care team comprised professionals from various disciplines, including genetics, clinical psychology, cardiology, ophthalmology, phoniatrics, and physical medicine and rehabilitation, in addition to specialists in palliative care. The diagnostic program encompassed genetic analysis predicated on medical and family history, pedigree evaluation, and counseling [23]. Clinical follow-up incorporated standardized assessments, including the Muscular Impairment Rating Scale (MIRS) [3,24]. A psychological evaluation was conducted to identify symptoms of anxiety or depression that potentially correlated with DM1 or its social implications. In accordance with these findings, targeted therapeutic interventions and longitudinal re-evaluations were provided. The rehabilitation therapy program included occupational therapy, physical therapy, speech–language therapy, phoniatrics therapy, and hydrotherapy.

For asymptomatic at-risk individuals, the pre-symptomatic protocol included an initial neuromuscular assessment, two genetic counseling sessions, and a minimum of three psychological screening interviews prior to genetic testing. The objective of these assessments was to ascertain the psychological readiness of the individual to receive the results of the genetic test. At the time of result disclosure, participants were informed of their potential carrier status and its implications for family members. Follow-up psychological evaluations were scheduled at one-week, four-week, six-month, and one-year intervals post-disclosure.

Pre-Natal Diagnosis (PND) for DM1 was conducted on DNA extracted from chorionic villus sampling (CVS) or amniotic fluid [25,26]. The diagnostic workflow followed the same procedures as postnatal symptomatic and pre-symptomatic testing. Maternal DNA was used to exclude maternal cell contamination, and in some cases, a sample from the unaffected parent was analyzed to confirm PCR results, particularly when the fetal alleles fell within the normal size range [27]. Couples undergoing PND were referred to the National Institute of Perinatology of Mexico (INPer) for pregnancy evaluation and maternal DNA sampling. Diagnostic testing was conducted at the INR-LGII. Genetic counseling and psychological support were provided throughout the pregnancy and during postnatal follow-up evaluations at one week, four weeks, six months, and one year after birth. Finally, Preimplantation Genetic Testing (PGT) was offered on a strictly voluntary basis and coordinated in collaboration with a private in vitro fertilization (IVF) clinic, in accordance with the guidelines established by the ESHRE PGT Consortium [28,29,30,31]. Nevertheless, the in vitro fertilization clinic was responsible for the clinical procedures. Continuous psychological and genetic support was provided by the INR-LGII.

During the enrollment phase, participants completed a structured interview. This interview covered motivations for genetic testing, expected outcomes based on potential results, information-sharing preferences, and the intended use of the genetic information.

### 2.3. Molecular Diagnosis

For diagnosis of all study subjects, genomic DNA was extracted from peripheral blood leukocytes using the Gentra Puregene Blood Kit (Qiagen, Hilden, NRW, Germany), according to the manufacturer’s instructions. This procedure was performed in INR-LGII. For PGT, the amniocentesis was performed at 16 weeks of gestation in INPer. The procedure involves withdrawing 15–20 mL of amniotic fluid from the amniotic cavity surrounding the fetus, using a small needle inserted through the abdomen under continuous ultrasound guidance. After DNA extraction from the amniotic fluid, the sample follows the same analytical steps as postnatal testing. Finally, for PGT, the embryo biopsy procedure was performed at the cleavage stage on day 3. Two blastomeres were removed from embryos that contained seven or more blastomeres, the isolation and DNA extraction was performed by private IVF, adhering to protocols that have been validated [28,29,30,31]. To assess normal and mildly expanded alleles (10–130 CTG repeats), fluorescent PCR was performed using 15 ng of genomic DNA as template and locus-specific primers flanking the CTG repeat: P1-Fw-FAM and P2-R (Accesolab, Mexico City, Mexico), as previously described by Warner et al. [32]. PCR amplification was carried out on an Applied Biosystems thermal cycler (Applied Biosystems, Foster City, CA, USA) under the following conditions: 35 cycles of 94 °C for 60 s (denaturation), 58 °C for 60 s (annealing), and 72 °C for 90 s (extension). For detection of larger expansions, triplet-primed PCR (TP-PCR) was employed using the fluorescently labeled P1 primer in combination with primers P3 and P4 to amplify multiple internal priming sites within the CTG tract, following protocols described by Warner et al. and optimized by Magaña et al. [32,33]. Thermocycling conditions for TP-PCR included 35 cycles of 94 °C for 60 s, 59 °C for 60 s, and 72 °C for 130 s. PCR products were mixed with deionized formamide and an internal size standard (ABI GeneScan-500 TAMRA Thermo Fisher Scientific, Waltham, MA, USA), denatured at 95 °C for 7 min, and rapidly cooled on ice for 5 min. Capillary electrophoresis was performed on an ABI PRISM 3730XL Genetic Analyzer (Applied Biosystems, Foster City, CA, USA) under the following conditions: 15 kV for 24 min at 60 °C with an injection time of 5 s. Fragment analysis and genotyping were performed using GeneScan V.3.1.2 software (Applied Biosystems). Samples from healthy individuals with apparent homozygosity (single allele) were re-analyzed via TP-PCR to detect potential undetected large expansions and exclude false negatives.

Subsequently, we randomly selected a proportional sample of 320 individuals with a confirmed DM1 diagnosis by TP-PCR who did not have an exact CTG repeat length previously determined by PCR and capillary electrophoresis. The sampling strategy ensured appropriate representation of all clinical phenotypes. This cohort was subsequently analyzed to determine the progenitor allele, allowing comparison across the different phenotypic groups. To estimate the size of large expanded alleles (progenitor alleles), Small-Pool PCR (SP-PCR) followed by non-radioactive Southern blotting was performed, as previously reported by Tomé S et al., [34]. This technique is based on the PCR amplification of a trinucleotide repeat from multiple small pools of input DNA, each containing approximately 0.5–200 genome equivalents. SP-PCR products were separated on agarose gels and transferred to nylon membranes for hybridization using digoxigenin (DIG)-labeled probes [34,35]. Detection was carried out via chemiluminescence on radiographic film. This approach enabled accurate sizing of alleles with >130 CTG repeats and allowed for assessment of somatic instability within individual samples.

### 2.4. Statistical Analysis

All analyses were conducted in Python 3.12 environment using specialized libraries optimized for each stage of data processing and modeling. Descriptive statistics (central tendency and dispersion) were calculated with Pandas (v2.2.1) software. Inferential tests, including normality assessments and group mean comparisons, were performed using SciPy (v1.11.4). Logistic regression with Statsmodels (v0.14.1). The risk of congenital myotonic dystrophy was estimated using logistic regression with Statsmodels (v0.14.1), with CTG repeat length serving as the primary predictor. The study’s participants were limited to individuals who carried pathogenic alleles; those with premutation carriers were excluded from the analysis. Congenital-onset cases were designated as events, while asymptomatic carriers were classified as non-events. Finally, the DM1-free survival was assessed using Kaplan–Meier estimates with age as the time scale, implemented via Lifelines (v0.27.8). Asymptomatic individuals at the last follow-up were censored, while symptom onset defined events. The survival curves and predictions from the Cox proportional hazards model were presented as DM1-free survival, which represents the cumulative probability of symptom onset by age. Group comparisons were performed using the Log-Rank test. Geospatial analyses were performed using Geopandas (v1.1.1), and visualizations were generated with Matplotlib (v3.10.3).

## 3. Results

### 3.1. Molecular Diagnosis of DM1 in Mexico

**Figure 1:**
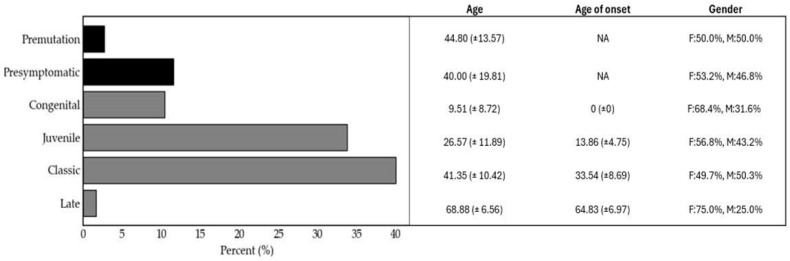
Frequency of carriers of expanded *DMPK* CTG repeats and their demographic characteristics. Pathological phenotypes and premutation status were determined following molecular diagnosis and clinical assessment. Mean age, age at onset, and sex distribution of the cohort are shown.

Patient evaluation was conducted at Mexico’s National Reference Center for neuromuscular diseases, the “INR-LGII” with the aim of implementing a diagnostic and clinical protocol for DM1. This protocol included the identification of individuals presenting clinical features suggestive of DM1 (index cases) through comprehensive clinical and molecular evaluation (symptomatic diagnosis), as well as pre-symptomatic diagnosis for the identification of at-risk relatives by constructing detailed family pedigrees. For 15 years, a total of 853 at-risk individuals were enrolled in the clinical protocol. Of these, 488 were confirmed as DM1-positive through molecular genetic testing. The distribution of clinical phenotypes in the studied cohort (Figure 1) revealed that the DM1 classic form was the most prevalent (40.16%), followed by the juvenile phenotype (33.40%). Conversely, congenital and mild phenotypes constituted smaller proportions (10.25% and 2.05%, respectively). This distribution underscores the high prevalence of early and classic onset symptomatic forms with multisystemic involvement, in agreement with previous clinical and genetic studies on DM1 [36]. All symptomatic patients were referred to diverse medical services for comprehensive care, including physical rehabilitation, cardiopulmonary rehabilitation, cardiology, neurology, neuropsychology, phoniatrics, audiology, and ophthalmology for continued medical and clinical follow-up.

Notably, the implementation of a combined symptomatic and presymptomatic diagnostic approach allowed the identification of 56 asymptomatic individuals (11.48%) who were carriers of a pathogenic allele. Finally, we identified 13 (2.66%) premutation carriers (Figure 1). Interestingly, most participants who completed the pre-symptomatic protocol reported multiple motivations for undergoing genetic testing. The mean age at time of testing was 38.26 years (range 18–88, SD 13.2 years). The primary reasons included: assessing the potential risk for their offspring (92.05%), preparing themselves both physically and psychologically to cope with the disease (81.58%), planning for the future (71.34%), alleviating uncertainty (53.76%), informing and preparing family members about the condition (52.09%), making informed decisions regarding family planning (40.79%), modifying their lifestyle (8.16%), employment-related considerations (8.60%), and guiding educational or career choices (7.32%). Following a psychological evaluation after a positive genetic test result, most carriers exhibited adequate emotional management. However, 20.08% showed some signs of emotional instability, 19.24% reported modifying their future plans, 17.99% experienced family conflicts, 7.11% underwent professional or occupational changes, and 5.2% reported marital dissolution. Additionally, 0.83% of participants expressed that the result had the potential to affect every aspect of their lives. In contrast, the majority of individuals (96.23%) indicated that they did not anticipate significant changes in the event of a negative result. Many of them reported feeling reassured and expressed increased optimism regarding their personal and professional lives. Only 4.38% anticipated potential emotional instability in the case of a negative outcome.

Molecular diagnosis is essential for informed reproductive decision-making; therefore, our team implemented both prenatal genetic diagnosis (PND) and preimplantation genetic testing (PGT) for Mexican patients. Although PND is available within public healthcare institutions, most affected couples opt to avoid pregnancy altogether or consider adoption. In our cohort, only three cases underwent prenatal testing, all of which were negative for the familial mutation, and thus no pregnancy termination was required. These pregnancies received continuous follow-up, including genetic and psychological counseling throughout the postnatal period. Families who elected PND reported high satisfaction, no emotional and psychological disturbances were identified during follow-up. In contrast, PGT is currently offered exclusively through private in vitro fertilization (IVF) clinics and remains inaccessible in public healthcare settings. Consequently, only one documented case of PGT was identified; the procedure was successful, resulting in an uncomplicated delivery at 39 weeks of gestation with an APGAR score of 9. Confirmatory testing in the newborn showed a heterozygous genotype within the normal allele range.

### 3.2. Socio-Epidemiologic Analysis

**Figure 2:**
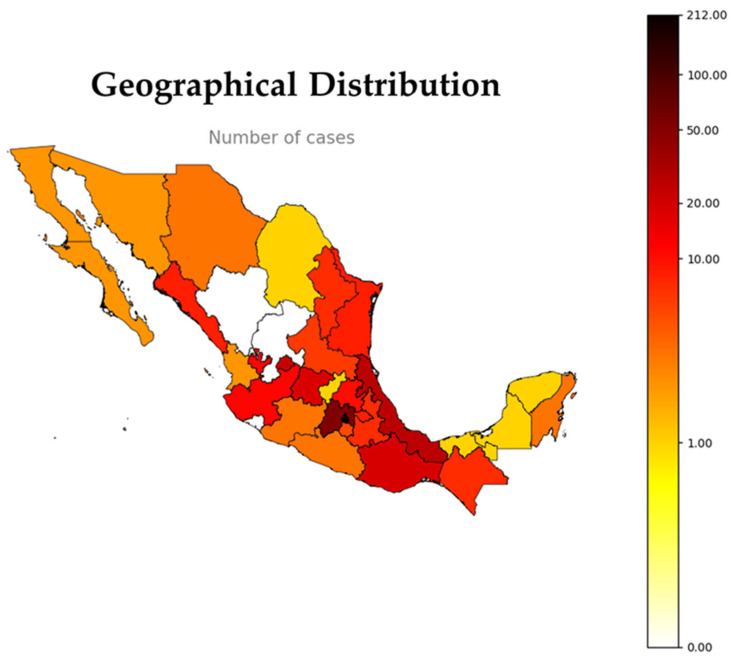
Heatmap illustrating the Geographical distribution of registered DM1 cases in Mexico. The map shows the absolute number of cases by federal entity, using a color scale ranging from white (no reported cases) to dark red (highest concentration, up to 212 cases).

The INR-LGII functions as a national reference center for neuromuscular disorders. It is the sole publicly accessible facility in Mexico that offers molecular diagnosis for DM1. Due to this central role, patients from both public and private healthcare institutions across the country are referred to the INR-LGII for confirmatory testing. Consequently, individuals with the capacity to travel to the capital are diagnosed at this facility, thereby facilitating nationwide diagnostic coverage. From 2010 to 2025, patients with DM1 were referred from 29 out of the 32 federal entities of Mexico, thereby emphasizing the wide geographic reach of the INR. The majority of cases were concentrated in central and southeastern states, with the highest burdens observed in Mexico City (46.90%), the State of Mexico (11.50%), Veracruz (5.75%), and Aguascalientes (5.09%) (Figure 2). Conversely, the northern and far southeastern regions documented a reduced number of diagnosed cases. This distribution may be indicative of both true variations in disease prevalence and disparities in access to specialized diagnostic centers and variations in healthcare infrastructure. Furthermore, population density and geographic proximity to the INR-LGII have been demonstrated to influence referral patterns and patient mobility.

Notably, access to pre-symptomatic diagnosis, requiring multiple follow-up visits for genetic counseling and psychological support, was predominantly concentrated in Mexico City and the State of Mexico (73.21%), while symptomatic diagnosis was frequently recorded exclusively in patients from distant states, where recurrent visits are not feasible. Figure 2 presents a geographic heatmap that integrates actual case counts with patterns of healthcare access and centralization of services, thereby highlighting the interplay between epidemiological burden and systemic healthcare factors. Furthermore, the capacity of patients to access diagnostic services, as well as to engage in follow-up and family-based studies, is significantly influenced by sociodemographic factors.

A comprehensive understanding of the social factors influencing DM1 care is provided by variables such as educational attainment, employment status, housing conditions, socioeconomic level, and access to a primary caregiver and the healthcare system (public or private) through which the patient was referred (see Table 1). These factors not only influence the accessibility of diagnostic services but also reflect broader structural inequities that impede timely medical intervention and family planning in genetically inherited diseases such as DM1.

With respect to educational attainment, the prevalence of illiteracy was found to be relatively low, and the proportion of individuals holding postgraduate degrees was observed to be limited. The majority of the participants (84.29%) had completed only basic education (elementary, junior high, or high school), while 12.57% had obtained a bachelor’s degree (see Table 1). This finding aligns with reports on the Mexican population, where only 4.2% of the population is illiterate, 83.92% has attained some level of basic education, and only 11.97% has completed higher education, defined as a bachelor’s degree or postgraduate studies [37]. This educational distribution among DM1 patients is reflected in employment status; 28.58% of participants reported current or past formal employment, and 24.87% were still pursuing academic studies, predominantly individuals with classic or mild disease phenotypes (Table 1). Nevertheless, the progression of disability in DM1 has had a substantial impact on work capacity. A survey revealed that 23.28% of patients were unemployed, while an additional 23.28% identified as homemakers. Conversely, at the national level, an unemployment rate of 2.8% was reported during the first half of 2025, suggesting that disability directly impacts employment opportunities and the social inclusion of affected individuals [38]. Consequently, 46.4% of the cohort lacked a stable income source, thereby underscoring the socioeconomic vulnerability associated with the disease (Table 1). These trends correspond with the socioeconomic status (SES) classifications utilized in Mexico’s public healthcare system to assess eligibility for services and associated costs, particularly among patients without formal social security coverage. SES classification encompasses a variety of factors, including but not limited to, household income, housing conditions, access to basic services, ownership of durable goods, and educational level. Patients are categorized on a scale ranging from level 0, which indicates extreme poverty and full subsidy, to level 6, which indicates high income (see Table 1). In this cohort, 34.89% of individuals were classified within the lowest SES brackets (levels 0 and 1), while 64.53% were identified as middle class (levels 2–4). A negligible percentage of the population, amounting to less than 1%, were classified within the uppermost SES categories, designated as levels 5–6. These findings are corroborated by housing data, which indicate that only 14.87% of patients resided in dwellings constructed with non-durable materials or those typically associated with poverty. This suggests that while educational and employment opportunities may be limited, the majority of patients reside in structurally adequate living environments. Despite the evident disability burden, only 32.7% of the cohort received governmental assistance for disability (see Table 1). This is in stark contrast to the 74.52% of patients who require daily support from a primary caregiver. Typically, these caregivers are close relatives (e.g., parents, spouses, or siblings) who often reduce their working hours or leave their jobs to provide individualized care (see Table 1). These dynamics further impact household income and economic resilience among families affected by DM1. Consequently, a mere 25.48% of patients retain complete autonomy in their daily activities, underscoring the pressing need for more robust social and healthcare support systems for this vulnerable population.

### 3.3. Clinical and Genetic Features in Patients with DM1

The age range of DM1 patients in our cohort ranged from birth to 75 years, with a mean age at onset of 34.08 ± 16.55 years (mean ± SD), a distribution consistent with previous reports in other populations [23,39,40,41]. To better delineate the clinical spectrum of the disease, patients were categorized into four phenotypic groups: congenital, juvenile, classic, and mild. Furthermore, an analysis of pre-symptomatic carriers and premutation subjects was incorporated to facilitate a more comprehensive understanding of early and subclinical manifestations. A summary of the most prevalent clinical features is provided in Table 2.

The presence of muscular weakness was observed in all patients (100%), with a predominance of facial and distal involvement across phenotypes. As anticipated, distal weakness manifested more frequently than proximal weakness across the entire cohort. The hallmark manifestation of myotonia was consistently observed in patients exhibiting juvenile and classic phenotypes, though its prevalence demonstrated variability in congenital and mild forms. Evidently, patients with the mild phenotype exhibit a preserved quality of life, as their life span is within the normal range. Most individuals present only slight symptoms, typically characterized by ptosis, myotonia (sustained muscle contraction) and muscle weakness, which is often misinterpreted as age-related sarcopenia. Additionally, cataracts are frequently reported in this group. In contrast, individuals with the classic and juvenile phenotypes, in addition to consistently exhibiting common features such as muscle weakness and wasting, myotonia, and frequent cardiac conduction abnormalities, also presented multiple systemic manifestations, these included dysarthria, hypersomnia, cataracts and gastrointestinal dysfunction (dysphagia and constipation). Notably, neuropsychiatric manifestations such as apathy, depression, and anxiety showed an age-dependent increase, reaching frequencies above 25% in the classic phenotype group. Mood alterations and avoidant personality traits were commonly observed in these individuals. Finally, the congenital subgroup displayed universal penetrance of muscular hypotrophy, ptosis, and facial weakness (100%), along with a high prevalence of intellectual disability (50%), whereas clinical myotonia was reported at a much lower frequency (20%). Unfortunately, this phenotype is highly severe, and both life expectancy and overall quality of life are markedly reduced. These findings underscore the multisystemic nature of DM1 and highlight the need for a multidisciplinary approach to patient care and monitoring, tailored to the phenotype-specific progression and burden of disease [42,43].

**Figure 3:**
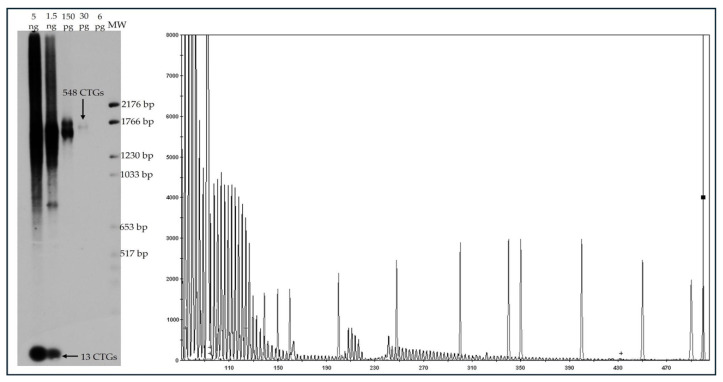
Detection of interruptions within the CTG repeat expansion in the *DMPK* gene. (**Left**): Southern blot analysis used to determine the number of CTG repeats. (**Right**): Electropherogram showing a characteristic pattern of a pathological expansion with interruptions. The gray signal denotes the fluorescent products generated by TP-PCR.

Molecular diagnosis was conducted using conventional PCR and triplet-primed PCR (TP-PCR) techniques. To further detect of progenitor allele expansions, we performed small-pool PCR (SP-PCR) in a representative subset of 320 patients across all phenotypes. The amplified products are separated by agarose gel electrophoresis and detected by Southern blot hybridization under conditions that permit the identification of products originating from single input molecules (Figure 3; Left panel). This approach enables detailed quantification of repeat-length variability within a sample, capturing both the most common variants (progenitor alleles) and rare alleles present in a limited proportion of cells. The molecular-weight marker scale (MW), displayed on the right, is converted into repeat-number units. At low DNA input concentrations, the progenitor alleles are clearly resolved, whereas at higher inputs, these predominant variants merge; however, this condition facilitates the detection of rare, very large expansions that occur only in a small subpopulation of cells. The observed range of CTG repeats in expanded progenitor DM1 alleles was 51 to 1138. A general pattern emerged linking repeat length with phenotypic severity. Congenital cases were typically associated with expansions > 900 CTG repeats, juvenile phenotypes with ~600, classic forms with ~350, and mild phenotypes with ~100 repeats (Table 2). These findings align with previously reported correlations between CTG repeat length and clinical expression of DM1 [11,39].

**Figure 4:**
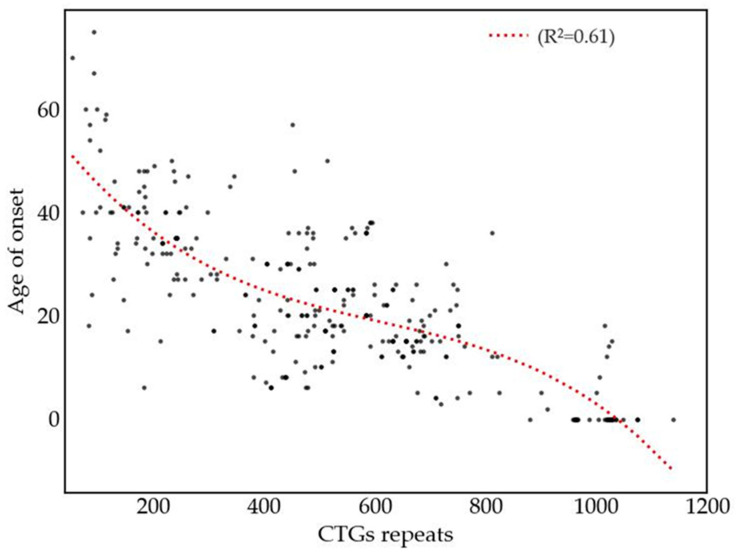
Relationship between CTG expansion and age at symptom onset in a group of DM1 patients (cohort of 320).

Despite interindividual variability, an inverse correlation was observed between repeat size and age of onset (Figure 4). A cubic regression model yielded an R^2^ value of 0.612, indicating a moderate association between expanded allele size and earlier symptom onset. This genotype-phenotype correlation supports earlier findings, including those by Morales et al. [12], who described how expansion size and the presence of repeat interruptions can modulate disease severity and clinical progression.

Of particular interest is the identification of 14 patients with repeated CTG tract interruptions, representing 2.82% of the DM1 carriers with positive diagnoses (Figure 3; right panel). The patients with interruptions belong to 10 families. Of the 14 patients with interruptions, three were found to have inherited the interruptions from their father, one from their mother, two apparently presented de novo inheritance, and the transmission of the remaining patients with interruptions could not be determined due to the absence of diagnoses in immediate relatives due to various causes, mainly deceased or for being foreign patients. According to the electrophoretic patterns obtained by TP-PCR, the interruptions were associated with apparent tri- or hexanucleotide motifs, with some alleles showing up to five interruptions (Figure 3; right panel). However, sequencing will be required to determine the exact composition of these interruptions. Interestingly, a clear delay in the onset of symptoms is evident, and although longitudinal follow-up is still lacking, a lower severity of signs and symptoms is perceived. Most individuals exhibit an improved quality of life and greater social inclusion.

### 3.4. Symptom-Free Survival Analysis and Modifying Effects of CTG Expansion Size and Interruptions

**Figure 5:**
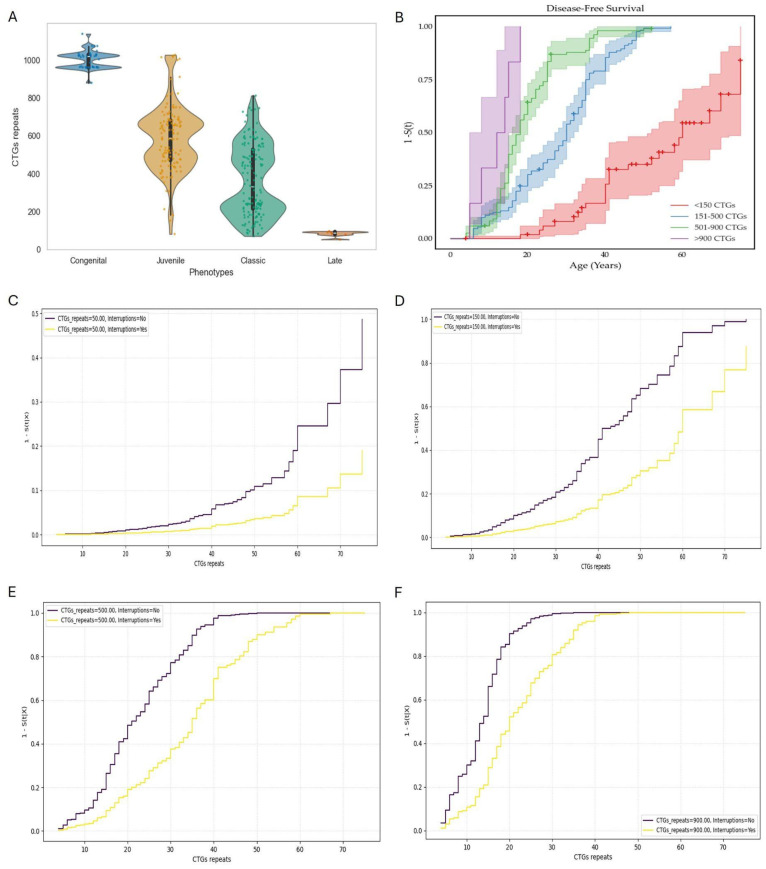
Genotype-phenotype correlation for the myotonic dystrophy-free period analysis and modifying effects of CTG expansion size and interruptions. (**A**) Correlation genotype-phenotype in the DM1 cohort. The jitter plot shows the distribution of CTG repeats according to the different phenotypes. (**B**) Disease-free survival curves estimated by the Kaplan–Meier method according to the size of the CTG expansion in DM1 patients. The predictions of the Cox proportional hazards model are presented as 1–DM1-free survival. (**C**–**F**) Survival analysis and modifying effects of CTG expansion size and interruptions. The predicted survival curves are shown stratified by interruptions at different CTG repeat thresholds. After confirming that the data followed a normal distribution, Student’s *t*-test revealed a statistically significant difference (*p* = 0.011). However, Student’s *t*-test applied to the CTG repetitions revealed no significant difference.

To further characterize the genotype–phenotype correlation within our DM1 cohort, a symptom-free survival analysis was conducted as a function of CTG repeat expansion size. As shown in Figure 5A, the clinical presentation exhibited a general correlation with the number of CTG repeats. Nevertheless, a considerable overlap among expansion sizes was observed across phenotypic categories, particularly between the classic and juvenile forms of the disease. To address this overlap, the cohort was stratified into four groups according to CTG repeat length, approximated to their respective clinical phenotypes: 50–150 repeats (red, mild phenotype); 151–500 repeats (blue, classic phenotype); 501–900 repeats (green, juvenile phenotype), and >900 repeats (purple, congenital phenotype). The resulting Kaplan–Meier survival curves revealed a clear inverse relationship between CTG repeat length and age at symptom onset. Individuals carrying >900 CTG repeats exhibited a significantly reduced symptom-free survival time (defined as the time elapsed prior to the onset of clinical manifestations; see Section 2) compared with individuals harboring smaller expansions (Figure 5B). It is worth noting that the risk of developing clinically apparent symptoms associated with DM1 increases abruptly beyond 200 CTG repeats, as shown in the Log (Hazard Ratio) model (see Supplementary Figure S1A). Moreover, when the CTG repeat length exceeds 700 repeats, there is a clear increase in the risk of presenting a congenital phenotype, as demonstrated by the logistic regression model [R^2^ = 0.812, *p* < 0.001] (see Supplementary Figure S1B). These findings are consistent with previous reports demonstrating that larger CTG expansions are associated with an earlier onset and more severe phenotypic expression of the disease [12,44].

Notably, within these expansion categories, patients harboring interruptions in the CTG tract were identified. Using a univariate Cox proportional hazards analysis, we determined that, in addition to CTG repeat expansion size, the presence of CTG interruptions also acts as a risk-modifying factor influencing symptom onset. The coefficient for the expanded allele was β = 0.004 (Wald *p* < 0.001), corresponding to a hazard ratio (HR) of 1.004 (95% CI: 1.004–1.005), indicating that each additional CTG repeat is associated with a 0.4% increase in the instantaneous risk of symptomatic onset.

The presence of interruptions showed a β coefficient of −0.816 (Wald *p* = 0.009), with an associated hazard ratio of 0.44 (95% CI: 0.24–0.81), suggesting a protective effect that delays disease onset. Furthermore, implementation of a Cox proportional hazards model with restricted cubic splines (RCS) for the CTG repeat variable revealed that patients carrying interrupted alleles exhibited a slower clinical progression, reflected in a prolonged symptom-free survival (Figure 5C–F). This protective effect was most evident in the lower expansion categories (50–150 repeats), where interrupted alleles were associated with a marked delay in symptom onset. The impact of interruptions appeared to decrease with increasing expansion size. In the >900 repeat group, although a reduction in symptom-free survival was observed, it was less pronounced compared to the other groups. These findings support the notion that interruptions within the CTG repeat tract act as a key genetic modifier of disease onset and progression [45,46]. Collectively, these data reinforce the utility of molecular stratification, not only for advancing the understanding of DM1 pathogenesis, but also for informing prognostic models and developing potential therapeutic interventions targeting repeat instability.

## 4. Discussion

### 4.1. Experiences and Challenges in the Diagnosis and Management of DM1

The present study provides an updated overview of a 15-year longitudinal investigation of DM1 in the Mexican population. This comprehensive analysis of an unusually large national cohort integrates multiple research areas, including molecular diagnosis, socio-epidemiological characterization, genotype–phenotype correlations, and the impact of genetic mutations on symptom-free survival, along with multidisciplinary clinical and psychological management. The long-term monitoring of this orphan disease within a Latin American population with a distinct ethnic background underscores the challenges of addressing rare disorders in settings where healthcare systems are still developing, and specialized care is available only in a few national reference centers The study of 853 at-risk individuals from 89 families identified 488 DM1 mutation carriers. The phenotypic distribution was consistent with previous reports, with the classic (40.2%) and juvenile (33.4%) forms being most frequent, followed by congenital (10.3%) and mild (2.1%) phenotypes. This supports DM1 being the most common adult-onset muscular dystrophy worldwide. Mild cases likely remain underdiagnosed, as carriers typically exhibit clinical manifestations only in advanced age and often lack detailed family histories. The male-to-female ratio was approximately 1:1 across most phenotypes, although congenital (2:1) and mild (3:1) forms were more common in males, supporting earlier observations of a male predominance [47].

The cohort of DM1 patients were diagnosed and counseled at the INR–LGII, the only public center in Mexico providing molecular diagnosis for this disorder. This setting explains the high proportion of mutation-positive cases, as presymptomatic testing requires genetic counseling and psychological evaluation, which limits participation mainly to individuals from Mexico City and surrounding areas. Moreover, both molecular diagnosis and multidisciplinary patient follow-up are exclusively provided at the national reference center located in the capital, further restricting access for individuals living outside this area. Although patients came from nearly all regions, most referrals originated from central and southeastern states (Mexico City, State of Mexico and Veracruz), reflecting population density and healthcare accessibility in these regions, as well as their geographical proximity to the reference center. Given this geographical and healthcare access bias, the accurate national prevalence of DM1 remains undetermined, as a significant proportion of patients in remote regions are likely to remain undiagnosed. This highlights the urgent need to decentralize diagnostic and specialized care services through technology transfer to other reference centers and specialized hospitals across the country. Moreover, the clinical management of DM1 requires a multidisciplinary approach, which poses an additional challenge since not all healthcare institutions possess the infrastructure or expertise to provide comprehensive care to patients and their families.

Despite the fact that the presymptomatic testing program was predominantly implemented in Mexico City and its metropolitan area, participation rates were found to be remarkably high. The observed rate of protocol adherence (~75%) and the rate of withdrawal (~22%) were comparable to the rates reported in international predictive testing programs for neuromuscular diseases conducted in various countries, thereby reflecting a high degree of acceptance of genetic testing within the population [48,49]. The majority of test requests were initiated during the first two years of implementation, driven by the experiences of early participants, their educational level, and the concentration of specialized services in the capital. The primary reasons for withdrawals from the study were mobility limitations or travel times. Additionally, psychological exclusion criteria were implemented to ensure safe testing conditions by screening for severe psychiatric disorders or high anxiety/depression. These results underscore the necessity of augmenting public awareness initiatives and establishing community-based genetic counseling services and training programs for medical professionals and psychologists at the national level. Such efforts could potentially improve pre-test understanding and adherence to program guidelines across various regions. The average age of the participants is very similar to international trends [50,51,52], suggesting that the individuals included in the study were of reproductive age and were motivated by family planning and health decision-making. The majority of the participants were in partnerships, and approximately half were childless prior to undergoing testing, thereby underscoring the significance of reproductive motivations in pursuing predictive testing.

With respect to PND and PGT, participation rates have remained low in recent years. Although PND is legally permitted in Mexico City, sociocultural, religious, and ethical factors often deter individuals from considering pregnancy termination, leading many to choose childlessness or adoption instead. On a national scale, legal and public health restrictions further limit access, as most states do not authorize such procedures. PGT, though potentially more acceptable, is inaccessible to most due to its absence in public healthcare and high private costs. These barriers are consistent with those observed in other Latin American populations, where cultural and legal constraints strongly influence reproductive decisions.

It is worth noting that the demographic characteristics of the DM1 cohort examined in this study are comparable to those of the Mexican population as a whole. There are no statistically significant differences in educational level, socioeconomic status, or household characteristics between the two groups [37,38]. This finding stands in contrast to the conclusions reported by Ganon et al., who indicated an association between DM1 and suboptimal educational attainment [18]. Consequently, these findings indicate that DM1 exhibits a uniform distribution across social strata. Nevertheless, underdiagnosis is probable among vulnerable populations with constrained access to healthcare. The disease’s progressive and disabling nature is well-documented. In our cohort, the unemployment rate was 23.3%, which is substantially higher than the national average of 2.8% in early 2025. Additionally, 23.3% of the cohort was engaged in household tasks. Furthermore, the obligations associated with caregiving can impede the employment prospects of certain relatives. Despite the existence of public disability assistance programs, only 32.7% of patients reported receiving such support, a finding that likely reflects limited awareness, bureaucratic barriers, or insufficient outreach. Therefore, the improvement of vocational rehabilitation services and the promotion of social inclusion could positively benefit individuals with DM1 by enhancing their functional capacity and overall quality of life.

### 4.2. From Clinical to Genetic Basis of DM1

A comprehensive clinical and molecular characterization of DM1 patients was conducted, and the patients were classified into congenital, juvenile, classic, and mild phenotypes based on age at onset and clinical presentation. The symptom profile exhibited marked consistency with those documented in other populations [15,47,53], with myotonia, distal muscle weakness, and gastrointestinal disorders being hallmark features [1,13]. Myotonia was observed to occur in a generalized manner within the classic and juvenile phenotypes. However, its occurrence was uncommon in congenital cases, with the exception of those cases in which the onset of symptoms had taken place at an advanced stage [54]. In contrast, patients with congenital forms of the condition exhibited a higher prevalence of facial weakness, ptosis, and intellectual disability [16,55,56] while symptoms in the mild phenotype were often subtle or clinically imperceptible [39,57]. Neuropsychiatric and behavioral disturbances are also commonly observed in patients with DM1 [53,58]. To precisely characterize the expanded CTG repeat alleles, we implemented small-pool PCR (SP-PCR) to determine the progenitor allele size [23,35]. The identification of the progenitor allele, defined as the allele carrying the CTG repeat expansion in the *DMPK* gene that is transmitted from either parent to the affected individual [12], is critical for these analyses. This allele functions as a direct reference point, thereby establishing the original expansion size prior to the occurrence of intergenerational instability or somatic variation. This observation is of particular pertinence in the context of DM1, a condition characterized by the presence of somatic mosaicism across different tissues [59,60]. Several studies have shown that CTG repeat length varies markedly across tissues, with greater instability in clinically affected tissues such as skeletal, cardiac, and smooth muscle, and more stability in the central nervous system [61,62,63]. Somatic instability is tissue-specific and increases with repeat size and age [64,65,66]. This variability likely reflects the influence of tissue-specific gene expression, DNA repair capacity, cis/trans-acting modifiers, and broader genomic variation, all of which may contribute to distinct clinical phenotypes [67,68]. It also remains unclear whether population genetic background impacts somatic mosaicism, underscoring the need for studies in diverse cohorts. In a subgroup of our 320 patients, analysis of the relationship between allele size and age at onset confirmed a strong non-linear association, fitting a cubic regression model (R^2^ = 0.612). This finding suggests that CTG repeat length contributes significantly to the variability in disease onset, corroborating prior research [12].

Notwithstanding, marked clinical and genetic heterogeneity was observed, suggesting the influence of modifying factors. In this manner, 14 patients (2.82%) exhibited interruptions within the CTG tract, consistent relatively with the reported 3–8% frequency of interrupted alleles in DM1 [45,46,69]. Although the cohort size was substantial, this frequency was slightly lower than that observed in several Caucasian populations, such as Italy (3.5%), the Czech Republic (5%), or the United Kingdom (8.4%) [69], which may be explained by population and geographical differences. Previous genomic analyses in DM1 patients from our region have demonstrated a distinct genetic background compared with European populations, with a higher genetic contribution from Native American ancestries [23]. Future comparisons with other Latin American populations may confirm whether population-related genetic factors influence the occurrence of these interruptions. While the clinical implications of these findings are still being investigated, the available data suggest that repeated interruptions in treatment may delay the onset of the disease and extend the period of survival without symptoms. Patients who did not have interruptions in their alleles showed more than twice the risk of early symptom manifestation compared with those who carried interrupted alleles. This finding is consistent with the results of the univariate Cox model, which indicated that both CTG repeat expansions and the presence of interruptions significantly influence the age at onset of DM1. Notably, CTG repeat interruptions have been observed to function as a protective genetic modifier, resulting in delayed disease onset and prolonged disease-free survival across various repeat ranges [70,71]. This protective effect is most pronounced in alleles with smaller expansions but persists, albeit to a lesser degree, in individuals carrying very large expansions (>900 repeats). Recent studies have proposed that CTG repeat interruptions may stabilize the expanded allele *in cis* by preventing the formation of abnormal secondary DNA structures, thereby reducing both somatic and germline instability. This attenuation limits RNA toxicity and is associated with a milder clinical phenotypic expression, including later age at onset and the absence of congenital forms [45,69,71]. In addition, the findings of this study align with prior reports that have documented the stabilizing effect of repeated interruptions in other trinucleotide repeat disorders, such as Fragile X syndrome and Huntington’s disease [72,73]. In these cases, the interventions have been shown to mitigate repeat instability, delay the onset of symptoms, and reduce the severity of the disease [73].

In DM1, the severity of the disease has traditionally been correlated with the size of the CTG repeat expansion and the age at which symptoms first appear. However, given the highly polymorphic nature of these dynamic mutations and the considerable variability in the timing of clinical identification across individuals, establishing clear genotype–phenotype correlations remain challenging. Consequently, the evaluation of disease-free survival, delineated as the period during which a mutation carrier exhibits no symptoms, offers a more objective metric for measuring disease progression [74]. The findings demonstrate a clear relationship between CTG repeat length and disease-free survival across approximated clinical phenotypes. Congenital cases exhibit the shortest disease-free survival, followed by juvenile and classic forms, whereas mild phenotypes display the longest symptom-free intervals. These observations underscore the efficacy of disease-free survival as a critical parameter for evaluating the natural history of DM1 and for monitoring potential therapeutic interventions in longitudinal studies.

While these findings are descriptive for the Mexican population, they provide critical insights for future clinical monitoring and epidemiological assessment of DM1. This condition, despite being classified as a rare disorder, may have a higher-than-expected prevalence in our population, as suggested by Murillo-Melo et al. [23].

## 5. Conclusions

In summary, although myotonic dystrophy type 1 (DM1) is considered a rare disease worldwide, a substantial number of cases have been identified in Mexico. The implementation of a comprehensive national program integrating molecular diagnosis, multidisciplinary clinical management, and long-term follow-up has led to substantial improvements in patient care and has expanded our understanding of the disease within a Latin American context.

The findings of our study offer significant insights into the epidemiological, socioeconomic, and clinical behavior of DM1 in the Mexican population. These insights demonstrate the feasibility and impact of molecular diagnosis at a national level. Furthermore, we characterized the relationship between the size of the progenitor CTG repeat allele and both the age at disease onset and disease-free survival, while highlighting the protective role of CTG repeat interruptions as genetic modifiers influencing disease expression.

The continued monitoring of this cohort will enable the identification of novel clinical and molecular features, the discovery of reliable biomarkers and therapeutic targets, and the development of personalized management strategies and emerging therapies tailored to the needs of Latin American populations.

## Figures and Tables

**Table 1:** Percentage distribution of sociodemographic variables, housing conditions, economic aid, socioeconomic level, and referral source in a cohort of patients with DM1.

	Category	Percentage (%)
Education	High school	29.84
	Junior high school	26.7
	Elementary school	14.66
	Bachelor’s degree	12.57
	Infancy/Childhood	9.95
	Adult with incomplete elementary school	3.14
	Postgraduate studies	1.57
	Adult with no formal education	1.57
Employment	Student	24.87
	Unemployed	23.28
	Homemaker	23.28
	Private employee	12.17
	Merchant	6.35
	Government employee	5.82
	Self-employed	2.65
	Retired	1.59
Housing Material	Permanent material	85.14
	Permanent and provisional materials	10.14
	Cardboard/wood/adobe sheet	4.73
Economic Aid	No assistance	67.3
	Received assistance	32.7
Socioeconomic Level	Level 0	6.4
	Level 1	28.49
	Level 2	26.16
	Level 3	30.23
	Level 4	8.14
	Level 5	0.58
	Level 6	0
Caregiver	Father	33.12
	Independent	25.48
	Mother	18.47
	Husband	12.74
	Brother	2.55
	Wife	2.55
	Grandparents	1.91
	Uncle/Ant	1.27
	Ex-partner	1.27
	Son	0.64

**Table 2:** Frequency of clinical manifestations by phenotype in symptomatic patients with myotonic dystrophy type 1 (DM1).

Clinical Characteristics					
	Congenital	Juvenile	Classic	Mild	Total
	(*n* = 50)	(*n* = 163)	(*n* = 196)	(*n* = 10)	(*n* = 419)
Myotonia	20.0%	100.0%	100.0%	20.0%	88.5%
Muscular weakness					
Generalized	50.0%	19.6%	41.8%	30.0%	33.8%
Upper-limb	75.0%	61.3%	86.2%	70.0%	74.8%
Lower-limb	50.0%	41.7%	86.2%	70.0%	64.2%
Proximal	50.0%	9.8%	25.0%	20.0%	22.0%
Distal	75.0%	67.5%	89.8%	80.0%	79.1%
Facial	100.0%	84.0%	83.2%	80.0%	85.4%
Ptosis	100.0%	58.3%	55.6%	60.0%	62.1%
Masseter	50.0%	45.4%	46.9%	50.0%	46.8%
Sternocleidomastoid	50.0%	12.9%	0.0%	10.0%	11.2%
Intercostal	0.0%	3.1%	0.0%	10.0%	1.4%
Claudicating gait	25.0%	22.7%	16.8%	20.0%	20.2%
Hypotrophy	100.0%	45.4%	52.5%	50.0%	55.3%
Cardiological disorders					
Conductions defects	0.0%	3.1%	2.5%	10.0%	2.6%
Tachyarrhythmias	0.0%	6.7%	0.0%	10.0%	2.8%
Neuropsychiatric alterations					
Evasive personality	0.0%	19.6%	11.2%	10.0%	13.1%
Depression	0.0%	28.8%	27.5%	30.0%	24.8%
Anxiety	0.0%	12.9%	8.2%	10.0%	9.1%
Intellectual disability	50.0%	0.0%	0.0%	10.0%	6.2%
Gastrointestinal disorders					
Dysphagia	0.0%	28.8%	19.4%	20.0%	20.8%
Diarrhea	0.0%	15.9%	5.6%	10.0%	9.0%
Constipation	25.0%	12.9%	8.2%	10.0%	12.1%
Megacolon	0.0%	3.1%	0.0%	10.0%	1.4%
Gallstones	0.0%	3.1%	8.2%	10.0%	5.3%
Others					
Frontal alopecia	50.0%	45.4%	50.0%	47.9%	48.2%
Dysarthria	75.0%	61.3%	55.6%	59.2%	60.2%
Previous pneumonia	0.0%	9.8%	8.2%	8.5%	7.9%
Nocturnal apneas	0.0%	35.6%	30.6%	31.0%	28.9%
Hypersominia	50.0%	77.3%	58.2%	66.2%	64.8%
**Molecular Analysis, CTG Repeats Determination (*n* = 330)**					
	**Congenital**	**Juvenile**	**Classic**	**Mild**	**Total**
	**(*n* = 25)**	**(*n* = 139)**	**(*n* = 156)**	**(*n* = 10)**	**(*n* = 330)**
Short allele	12.44 (±3.90)	12.56 (±5.00)	11.50 (±4.013)	10.00 (±6.00)	11.96 (±4.31)
Expanded allele	1002 (±47.18)	586.91 (±173.76)	359.47 (±189.17)	95 (±26.64)	464.31 (±293.17)

Clinical characteristics were evaluated only in symptomatic patients (419 patients with DM1). The assessment of CTG repeats was based on data obtained by PCR, with a detection limit of 130 CTG repeats (mild cases), and in selected patients, it was further characterized by SP-PCR.

## Data Availability

The original contributions presented in this study are included in the article/Supplementary Material. Further inquiries can be directed to the corresponding author(s).

## Supplementary Materials

The following supporting information can be downloaded at https://www.mdpi.com/article/10.3390/genes16121515/s1: Supplementary Figure S1. Effect of CTG repeat length on age at symptom onset and risk of the congenital form in DM1. (S1A) The effect of CTG repeat length on age at symptom onset is shown as the logarithm of the hazard ratio (log[HR]) with 95% confidence intervals, estimated using a Cox proportional hazards regression model. (S1B) Predicted risk of congenital myotonic dystrophy according to CTG repeat length. The solid black line represents the estimated probability, and the shaded gray area indicates the 95% confidence interval. Colored dashed lines denote predefined risk thresholds: green (1%), blue (50%), and purple (99%) with their corresponding confidence intervals.
