# Supplementary figures and images for: Fifteen Years of Myotonic Dystrophy Type 1 in Mexico: Clinical, Molecular, and Socioeconomic Insights from a National Reference Cohort
Source: Genes (Basel). 2025 Dec 17;16(12):1515. doi: 10.3390/genes16121515 (PMC12732758; doi:10.3390/genes16121515)

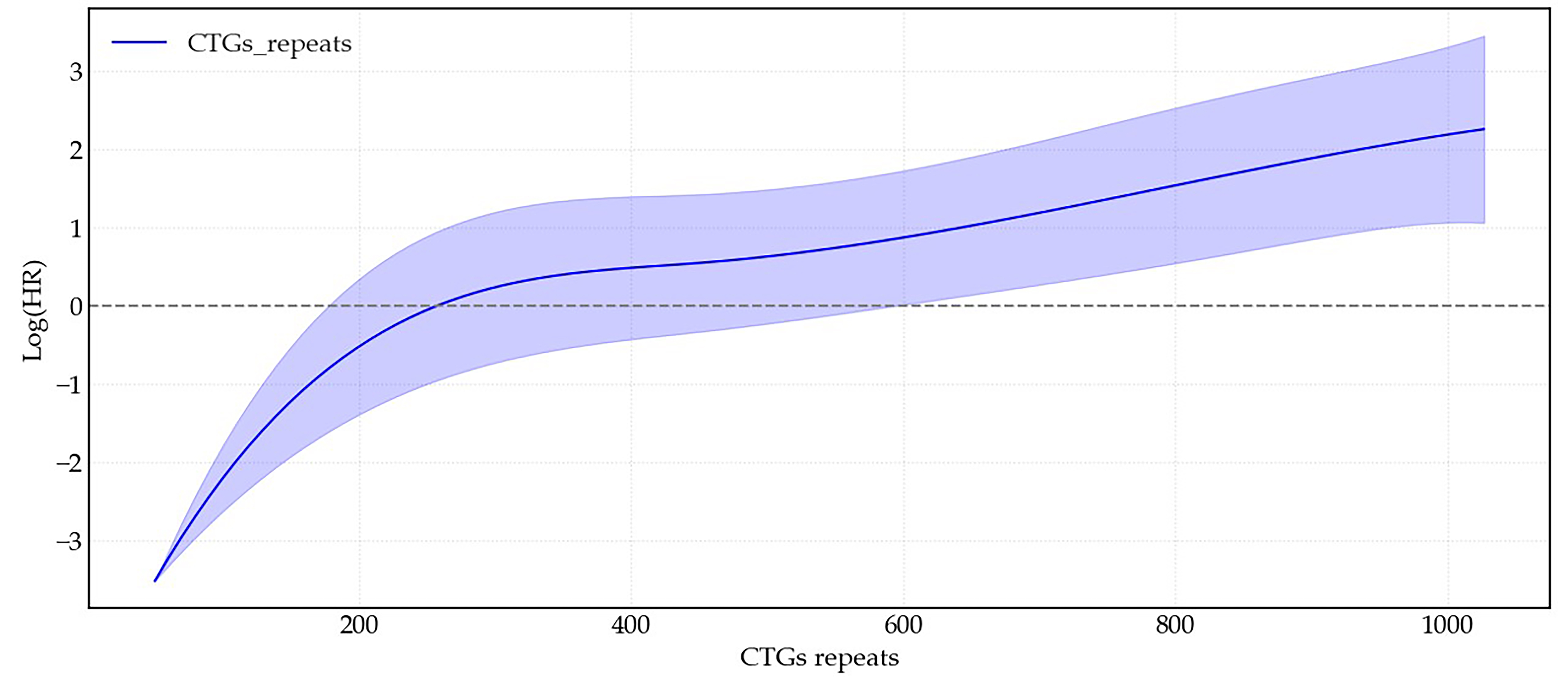

Supplement: Supplementary file 1 [file genes-16-01515-s001.zip › Sup S1A 600.jpg]

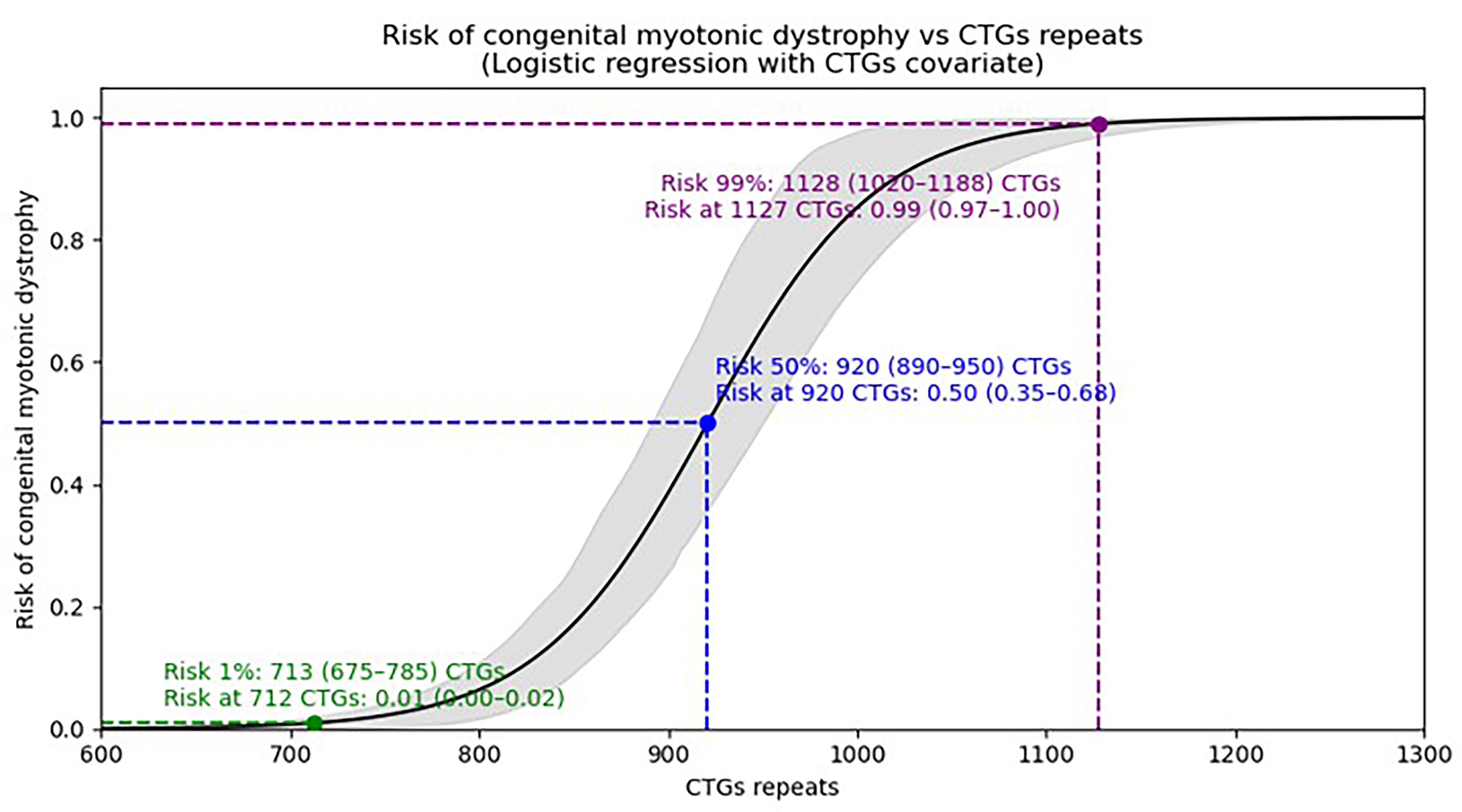

Supplement: Supplementary file 1 [file genes-16-01515-s001.zip › Sup S1B 600.jpg]
